# Oxidant and Antioxidant Levels in Different Stages of Chronic Kidney Disease

**DOI:** 10.7759/cureus.102115

**Published:** 2026-01-22

**Authors:** Pallavi Sagar, Ravi Shekhar, Kumar Pranay, Prit P Singh, Praveen Kumar

**Affiliations:** 1 Biochemistry, All India Institute of Medical Sciences, Patna, Patna, IND; 2 Biochemistry, Indira Gandhi Institute of Medical Sciences, Patna, IND; 3 Nephrology, Indira Gandhi Institute of Medical Sciences, Patna, IND; 4 General Medicine, Indira Gandhi Institute of Medical Sciences, Patna, IND

**Keywords:** antioxidant status, chronic kidney disease, lipid peroxidation, malondialdehyde, oxidative stress, superoxide dismutase

## Abstract

Background

Chronic kidney disease (CKD) is a progressive renal disorder strongly associated with oxidative stress, which contributes to renal damage and systemic complications. Oxidative stress results from an imbalance between the production of reactive oxygen species (ROS) and antioxidant defence mechanisms. This study aimed to evaluate oxidant and antioxidant levels across different stages of CKD.

Methods

This cross-sectional comparative study was conducted on 180 subjects, including 90 CKD patients and 90 healthy controls. CKD patients were classified into Stage III, Stage IV, and Stage V. Serum malondialdehyde (MDA) and superoxide dismutase (SOD) were measured to assess oxidative stress using standard laboratory assays. Data were analysed using one-way ANOVA with Brown-Forsythe adjustment, and a p-value <0.05 was considered statistically significant.

Results

A progressive increase in serum MDA and a significant decrease in SOD levels were observed across CKD stages compared with controls. Stage V patients demonstrated the highest MDA concentrations and the lowest SOD activity, indicating severe oxidative imbalance. Differences between groups were statistically significant (p < 0.001). Additionally, serum creatinine levels showed a consistent rising trend with advancing CKD severity.

Conclusion

The study findings indicate a shift toward oxidant dominance as CKD progresses, characterised by enhanced lipid peroxidation and reduced antioxidant enzyme activity. These biochemical alterations highlight the importance of oxidative stress monitoring in CKD and suggest the potential benefit of antioxidant-targeted therapeutic strategies. Further longitudinal and interventional research is necessary to explore the clinical impact of oxidative stress reduction on CKD outcomes.

## Introduction

Chronic kidney disease (CKD) is a progressive disorder defined by abnormalities of kidney structure or function persisting for more than three months, resulting in irreversible loss of nephrons and declining glomerular filtration rate (GFR) [1]. It affects millions worldwide and remains a major global health burden associated with substantially increased cardiovascular morbidity and mortality [2,3]. Multiple mechanisms contribute to renal deterioration in CKD, among which oxidative stress has been identified as a key pathological factor [4].

Oxidative stress arises from an imbalance between excessive production of reactive oxygen species (ROS) and reduced efficiency of antioxidant defence systems [5]. Increased ROS in CKD leads to lipid peroxidation, DNA injury, and protein degradation, thereby accelerating cellular damage and fibrosis in renal tissue [6]. Malondialdehyde (MDA), a final product of lipid peroxidation, serves as a reliable biomarker of oxidative injury, whereas superoxide dismutase (SOD) is an essential enzymatic antioxidant responsible for converting superoxide radicals into harmless molecules [7]. Previous studies demonstrate a progressive rise in MDA and reduction in SOD with advancing CKD severity, indicating worsening oxidative imbalance [8-10].

Elevated oxidative stress and diminished antioxidant capability contribute to inflammation, endothelial dysfunction, and cardiovascular complications in CKD, which remain primary determinants of poor prognosis [11]. Therefore, understanding oxidant-antioxidant imbalance may guide therapeutic approaches targeting antioxidant supplementation or lifestyle modifications to slow disease progression and improve outcomes [12].

The present study was designed to evaluate oxidative stress in patients with CKD stages III, IV, and V, using serum MDA and SOD as biomarkers, and to compare the findings with those of healthy controls. This study adds valuable evidence supporting the progressive increase of oxidative stress with advancing CKD stages and underscores the clinical importance of early intervention.

## Materials and methods

This was a cross-sectional comparative study conducted from November 2022 to September 2023 at the Department of Biochemistry in collaboration with the Department of Nephrology and General Medicine at the Indira Gandhi Institute of Medical Sciences (IGIMS), Patna, Bihar, India. The study was approved by the Institutional Ethics Committee, IGIMS (approval number: 729/IEC/IGIMS/2022), and adhered to the Declaration of Helsinki and Good Clinical Practice guidelines. Written informed consent was obtained from all participants before enrollment.

Eligibility criteria

Inclusion Criteria

Participants aged 18 years and above who were clinically diagnosed with CKD Stages III, IV, and V, as defined by thresholds of estimated glomerular filtration rate (eGFR), who consented to participation, were included in the study. Healthy volunteer participants who were without kidney disease and chronic systemic illness were included as controls.

Exclusion Criteria

If, in the week prior to the study, a patient was documented as having an episode of acute kidney injury, active infection, febrile illness, autoimmune/chronic inflammatory disease, or hepatic or malignant disorders, or if they were taking any supplements or medications known to be antioxidants or to otherwise influence parameters of oxidative stress, they were excluded. Those participants who were receiving any treatment likely to affect oxidative biomarkers were also excluded.

Sample size calculation

The sample size was calculated using the formula for comparing means between two groups:

$n = \frac{2 \times \sigma^{2} \times (Z_{\alpha/2} + Z_{\beta})^{2}}{d^{2}}$

Given a confidence level of 95% (Z = 1.96), an 80% power (Z = 0.84), standard deviation (SD) based on previous studies on oxidative stress in CKD (σ = 1.1 for MDA) [6], and a 0.5 unit difference for the minimum detectable difference, the minimum sample size for each group was 45. Hence, 180 subjects (90 CKD patients, 90 healthy controls) were included to enhance statistical robustness.

Study cohort

A total of 180 subjects participated in the study, consisting of 90 clinically confirmed CKD patients and 90 healthy controls. All individuals were above 18 years of age. The CKD group was subdivided into Stage III, Stage IV, and Stage V based on standard clinical criteria, including eGFR and duration of kidney dysfunction according to Kidney Disease: Improving Global Outcomes (KDIGO) guidelines [1]. Each subgroup consisted of an equal distribution of subjects to allow meaningful comparison. All CKD Stage V patients included in this study were pre-dialysis, and none were undergoing hemodialysis at the time of sample collection.

Sample collection procedure

Venous blood samples were collected from each participant under strict aseptic conditions following an overnight fast of 10-12 hours. Samples were allowed to clot and then centrifuged to separate serum. The serum was stored under controlled temperature conditions until biochemical analysis was performed. All procedures were carried out using calibrated laboratory instruments and standard operating protocols to ensure accuracy and reproducibility. 

Biochemical estimation

Serum creatinine levels were analysed using Jaffe’s colorimetric method to assess renal function. Oxidative stress was evaluated using established biochemical markers. Serum MDA levels were measured by the thiobarbituric acid reactive substances (TBARS) assay, which quantifies lipid peroxidation through the formation of a pink chromogen measurable at 532 nm [13]. SOD activity was assessed using the Marklund and Marklund method [14], based on inhibition of pyrogallol autoxidation, and enzymatic activity was expressed in units/mL of serum.

All biochemical assays were performed according to standard laboratory guidelines with internal quality control monitoring. Internal quality control procedures, including duplicate sample analysis and calibration with standard controls, were applied to ensure accuracy and reproducibility of results. Correlation analysis was performed to assess the relationship between serum MDA levels and SOD activity. Correlation analyses involving metabolic confounders such as body mass index and lipid profile parameters were not included, as the primary objective of the study was to evaluate oxidative stress and antioxidant status across different stages of chronic kidney disease.

Statistical analysis

The collected data were tabulated and analysed using statistical software IBM SPSS Statistics for Windows, Version 26.0 (IBM Corp., Armonk, New York, United States). Results for continuous variables were expressed as mean ± SD. One-way ANOVA with Brown-Forsythe adjustment was applied to compare oxidative stress parameters across control and CKD subgroups. Post-hoc analysis was performed where applicable. A p-value less than 0.05 was considered statistically significant to determine meaningful variation between groups.

## Results

A total of 180 subjects were included in the study, comprising 90 clinically diagnosed CKD patients and 90 healthy controls. CKD patients were categorised into three groups based on disease severity: Stage III, Stage IV, and Stage V. 

Demographic profile

The demographic characteristics of the study population showed no major difference in mean age or gender distribution between healthy controls and CKD groups, indicating effective matching between comparison groups (Table 1).

**Table 1:** Demographic characteristics of study participants CKD: chronic kidney disease

Parameter	Healthy Controls (n=90)	CKD Stage III (n=30)	CKD Stage IV (n=30)	CKD Stage V (n=30)
Mean Age (years)	49.2 ± 6.8	50.6 ± 7.3	51.4 ± 6.9	52.8 ± 8.2
Male:Female	52:38	17:13	18:12	19:11

Renal function parameter

Serum creatinine levels demonstrated a progressive rise from CKD Stage III to Stage V, indicating worsening renal clearance capability. Mean serum creatinine levels are given in Table 2.

**Table 2:** Serum creatinine levels across groups One-way ANOVA (F = 1807.32, p < 0.001). Post-hoc Tukey test performed for pairwise comparison vs Control. Reference range: 0.6–1.2 mg/dL. CKD: chronic kidney disease

Study Group	Serum creatinine (mg/dL), mean±SD	p-value vs Control
Control	0.92 ± 0.13	–
CKD Stage III	2.38 ± 0.41	<0.001
CKD Stage IV	4.62 ± 0.58	<0.001
CKD Stage V	7.85 ± 0.77	<0.001

SOD activity

A statistically significant decline in SOD activity was observed in CKD patients relative to healthy controls. Stage V patients displayed the most pronounced reduction, reflecting impaired antioxidant defence mechanisms. Mean serum SOD levels are given in Table 3.

**Table 3:** Mean serum SOD levels across groups ANOVA (F = 439.51, p < 0.001). Post-hoc Tukey test performed for pairwise comparison vs Control. Reference range: 5–10 U/mL. SOD: superoxide dismutase; CKD: chronic kidney disease

Study Group	Serum SOD (U/mL), mean ± SD	p-value vs Control
Control	6.85 ± 0.72	–
CKD Stage III	5.12 ± 0.68	<0.001
CKD Stage IV	4.01 ± 0.55	<0.001
CKD Stage V	2.76 ± 0.49	<0.001

MDA levels

MDA values demonstrated a reverse pattern compared to SOD. CKD patients showed greater lipid peroxidation, which increased proportionally with CKD severity. Table 4 gives the mean serum MDA levels.

**Table 4:** Serum MDA levels across the groups One-way ANOVA (F = 508.74, p < 0.001). Post-hoc Tukey test performed for pairwise comparison vs Control. Reference range: 1–3 nmol/mL MDA: malondialdehyde; CKD: chronic kidney disease

Study Group	Serum MDA (nmol/mL), mean ± SD	p-value vs Control
Control	2.15 ± 0.41	–
CKD Stage III	3.86 ± 0.52	<0.001
CKD Stage IV	4.92 ± 0.66	<0.001
CKD Stage V	6.28 ± 0.71	<0.001

Correlation between SOD activity and MDA levels

Correlation analysis was performed to evaluate the relationship between antioxidant status and lipid peroxidation. Serum MDA levels showed a statistically significant inverse correlation with SOD activity among the study participants. As SOD activity declined, a corresponding increase in MDA concentrations was observed, indicating enhanced oxidative stress with reduced antioxidant defence.

Pearson’s correlation analysis demonstrated a strong negative correlation between SOD and MDA levels (r = -0.78, p < 0.001). This inverse relationship was consistently evident across all stages of CKD, with progressively increasing MDA levels corresponding to decreasing SOD activity.

## Discussion

The present study demonstrated that oxidative stress significantly increases with the progression of CKD, as evidenced by the progressive elevation in serum MDA and the corresponding reduction in SOD levels from Stage III to Stage V. These observations are consistent with growing evidence indicating that oxidative stress plays a crucial role in the pathogenesis and progression of CKD, contributing to cellular injury and systemic complications [15]. Increased MDA levels reflect lipid peroxidation and damage to cell membranes, which may result from the accumulation of ROS due to impaired renal clearance and chronic systemic inflammation [16].

The marked decline in SOD activity across CKD stages in this study indicates deterioration in enzymatic antioxidant defence mechanisms. Reduced SOD levels have been associated with impaired ability to neutralise superoxide radicals, leading to increased oxidative burden and tissue injury [17]. This imbalance between oxidants and antioxidants can trigger mitochondrial dysfunction and endothelial damage, accelerating renal fibrosis and contributing to cardiovascular complications, which remain the leading cause of mortality in advanced CKD stages [18]. The inverse relationship between SOD and MDA in our findings supports the oxidative-antioxidative shift associated with CKD progression.

Similar trends have been reported by earlier studies in haemodialysis and non-dialysis CKD patients, demonstrating elevated lipid peroxidation products and reduced activity of antioxidant enzymes [19]. Additionally, research suggests that therapeutic interventions aimed at reducing oxidative stress, such as antioxidant supplementation, nutritional strategies, and regular controlled exercise, may improve oxidative profiles in CKD patients and enhance clinical outcomes [20,21]. However, evidence regarding long-term benefits remains limited and requires extensive randomized controlled trials.

The clinical significance of this study lies in the potential utility of oxidative stress biomarkers such as MDA and SOD as indicators of renal disease progression and as prognostic markers for monitoring therapeutic outcomes. Incorporating oxidative stress evaluation into routine CKD assessment may assist clinicians in identifying patients at greater risk of advanced complications, including cardiovascular events [22]. 

There are some limitations for this research as well. It is a cross-sectional study, which does not allow for the ability to establish a cause-and-effect relationship between the oxidative stress markers and the progression of CKD. Though the sample size was sufficient, it was taken from a single tertiary center, which could limit the generalizability of the study. There were only two markers of oxidative stress that were analyzed: MDA and SOD. It would be more informative to evaluate a wider variety of both the inflamed biomarkers and the antioxidant ones. Finally, the factors that were not controlled, like the dietary intake of antioxidants, electrolytic physical activity, history of medications, dialysis status, and others, are some that could help determine what the oxidative stress levels would be.

Overall, the findings of the present study reinforce the role of oxidative stress in CKD progression and emphasise the importance of developing targeted antioxidant-based therapeutic strategies to slow disease advancement and improve patient survival.

## Conclusions

The findings of this study demonstrate a clear and progressive imbalance between oxidant and antioxidant levels in patients with CKD. Serum MDA levels increased significantly, while SOD activity decreased notably with advancing CKD stages, indicating rising oxidative stress and diminished antioxidant defence. The greatest deviation from normal values occurred in Stage V patients, reflecting severe oxidative burden associated with end-stage renal impairment. These results reinforce the role of oxidative stress in CKD pathophysiology and highlight the potential utility of MDA and SOD as biochemical indicators for disease severity assessment. The outcomes suggest that antioxidant-based interventions and strategies targeting oxidative stress may offer therapeutic benefit in slowing renal deterioration and reducing associated complications. Further research involving larger and longitudinal cohorts is recommended to validate these findings and explore intervention effectiveness.

## References

[1] (2024). KDIGO 2024 clinical practice guideline for the evaluation and management of chronic kidney disease. Kidney Int.

[2] Jha V, Garcia-Garcia G, Iseki K (2013). Chronic kidney disease: global dimension and perspectives. Lancet.

[3] Go AS, Chertow GM, Fan D, McCulloch CE, Hsu CY (2004). Chronic kidney disease and the risks of death, cardiovascular events, and hospitalization. N Engl J Med.

[4] Cachofeiro V, Goicochea M, de Vinuesa SG, Oubiña P, Lahera V, Luño J (2008). Oxidative stress and inflammation, a link between chronic kidney disease and cardiovascular disease. Kidney Int Suppl.

[5] Small DM, Coombes JS, Bennett N, Johnson DW, Gobe GC (2012). Oxidative stress, anti-oxidant therapies and chronic kidney disease. Nephrology (Carlton).

[6] Dounousi E, Papavasiliou E, Makedou A (2006). Oxidative stress is progressively enhanced with advancing stages of CKD. Am J Kidney Dis.

[7] Cherian DA, Peter T, Narayanan A, Madhavan SS, Achammada S, Vynat GP (2019). Malondialdehyde as a marker of oxidative stress in periodontitis patients. J Pharm Bioallied Sci.

[8] Raju DS, Lalitha DL, Kiranmayi P (2013). A study of lipid profile and lipid peroxidation in chronic kidney disease with special reference to hemodialysis. J Clin Res Bioeth.

[9] Liakopoulos V, Roumeliotis S, Bozikas A, Eleftheriadis T, Dounousi E (2019). Antioxidant supplementation in renal replacement therapy patients: is there evidence?. Oxid Med Cell Longev.

[10] Sagar P, Pranay K, Shekhar R, Singh PP, Kumar P (2023). Serum malondialdehyde in different stages of chronic renal disorder. Asian J Med Sci.

[11] Rapa SF, Di Iorio BR, Campiglia P, Heidland A, Marzocco S (2019). Inflammation and oxidative stress in chronic kidney disease — potential therapeutic role of minerals, vitamins and plant-derived metabolites. Int J Mol Sci.

[12] Jun M, Venkataraman V, Razavian M (2012). Antioxidants for chronic kidney disease. Cochrane Database Syst Rev.

[13] Ohkawa H, Ohishi N, Yagi K (1979). Assay for lipid peroxides in animal tissues by thiobarbituric acid reaction. Anal Biochem.

[14] Marklund S, Marklund G (1974). Involvement of the superoxide anion radical in the autoxidation of pyrogallol and a convenient assay for superoxide dismutase. Eur J Biochem.

[15] Vida C, Oliva C, Yuste C (2021). Oxidative stress in patients with advanced CKD and renal replacement therapy: the key role of peripheral blood leukocytes. Antioxidants (Basel).

[16] Verma S, Singh P, Khurana S (2021). Implications of oxidative stress in chronic kidney disease: a review on current concepts and therapies. Kidney Res Clin Pract.

[17] Aziz MA, Majeed GH, Diab KS, Al-Tamimi RJ (2016). The association of oxidant-antioxidant status in patients with chronic renal failure. Ren Fail.

[18] Ling XC, Kuo KL (2018). Oxidative stress in chronic kidney disease. Ren Replace Ther.

[19] Massy ZA, Nguyen-Khoa T (2002). Oxidative stress and chronic renal failure: markers and management. J Nephrol.

[20] Zhao M, Xiao M, Tan Q, Lyu J, Lu F (2023). The effect of aerobic exercise on oxidative stress in patients with chronic kidney disease: a systematic review and meta-analysis with trial sequential analysis. Ren Fail.

[21] Molina P, Vizcaíno B, Huarte E, Pallardó LM, Carrero JJ (2022). Nutritional disorders in chronic kidney disease: pathophysiology, detection, and treatment. Evidence‐Based Nephrology.

[22] Putri AY, Thaha M (2014). Role of oxidative stress on chronic kidney disease progression. Acta Med Indones.

